# Effectiveness of a new triple-row circular stapler in reducing the risk of colorectal anastomotic leakage: A historical control and propensity score–matched study

**DOI:** 10.1097/MD.0000000000029325

**Published:** 2022-07-08

**Authors:** Junichi Mazaki, Kenji Katsumata, Tetsuo Ishizaki, Noritoshi Fukushima, Ryutaro Udo, Tomoya Tago, Kenta Kasahara, Hiroshi Kuwabara, Masanobu Enomoto, Yuichi Nagakawa, Akihiko Tsuchida

**Affiliations:** a Department of Gastrointestinal and Pediatric Surgery, Tokyo Medical University, Tokyo, Japan; b Department of Preventive Medicine and Public Health, Tokyo Medical University, Tokyo, Japan.

**Keywords:** anastomotic leakage, circular stapler, double stapling technique, propensity score analysis, tri-staple

## Abstract

Anastomotic leakage (AL) after colorectal surgery is a serious complication. This study aimed to evaluate the effectiveness of the EEA™ circular stapler, a new triple-row circular stapler (TCS), relative to a conventional, double-row circular stapler (DCS).

A total of 285 patients who underwent anastomosis with the double stapling technique at the Tokyo Medical University Hospital between 2017 and 2021 were included in this nonrandomized clinical trial with historical controls using a propensity score (PS) analysis. The primary endpoint was the risk of AL.

We performed a 1:2 PS matching analysis. Before case matching, AL occurred in 15 (7.4%) and 2 (2.4%) patients in the DCS and TCS groups, respectively, with no significant difference (*P* = .17). After case matching, AL occurred in 13 patients (11.6%) and 1 patient (1.8%) in the DCS and TCS groups, respectively, revealing a significant difference (*P* = .04). Cox models were created by applying PS to adjust for group differences via regression adjustment. Odds ratios for AL in the DCS group versus the TCS group were 0.31 (95% confidence interval [CI]: 0.07–1.38) in the entire cohort, 0.15 (95% CI: 0.02–0.64) in the regression adjustment cohort, and 0.14 (95% CI: 0.02–1.09) in the 1:2 PS-matched cohort.

PS analysis of clinical data suggested that the use of TCS contributes to a reduced risk of AL after colorectal anastomosis CTwith the double stapling technique.

## 1. Introduction

Postoperative colorectal anastomotic leakage (AL) is a devastating complication^[1,2]^ that contributes not only to postoperative morbidity and mortality, but also to local recurrence and poor functional outcomes.^[3]^ A successful anastomosis and subsequent healing depend on several factors, including the tension between the 2 connected portions of the gastrointestinal tract, a healthy blood supply to surrounding tissues, and the mechanical strength of the formed anastomosis.^[4–6]^

The use of circular stapling devices to facilitate colorectal anastomosis was first described in the 1970s. This approach is now a standard practice and has consistently demonstrated equivalent safety and efficacy to hand-sewn anastomosis, with added advantages of shorter anastomotic time and greater reproducibility.^[7–9]^ The choice of surgical instruments greatly affects the safety of anastomotic operations from the viewpoint of mechanical strength and a healthy blood supply. Major advances have been made in the development of medical devices for rectal surgery, in particular, for rectal anastomosis with the double stapling technique (DST). A new circular stapler having a triple row of staples is expected to improve pressure resistance, as compared to the conventional, double-row circular stapler (DCS). However, the effectiveness of the new triple-row circular stapler (TCS) in reducing the risk of AL has not been fully examined or demonstrated.

The present study aimed to compare the clinical effectiveness of TCS (EEA™ circular stapler with Tri-Staple™ technology, 28 mm Medium/Thick, Covidien, New Haven, CT) and DCS using a propensity score analysis.

## 2. Materials and Methods

### 2.1. Patients

**Figure 1.**
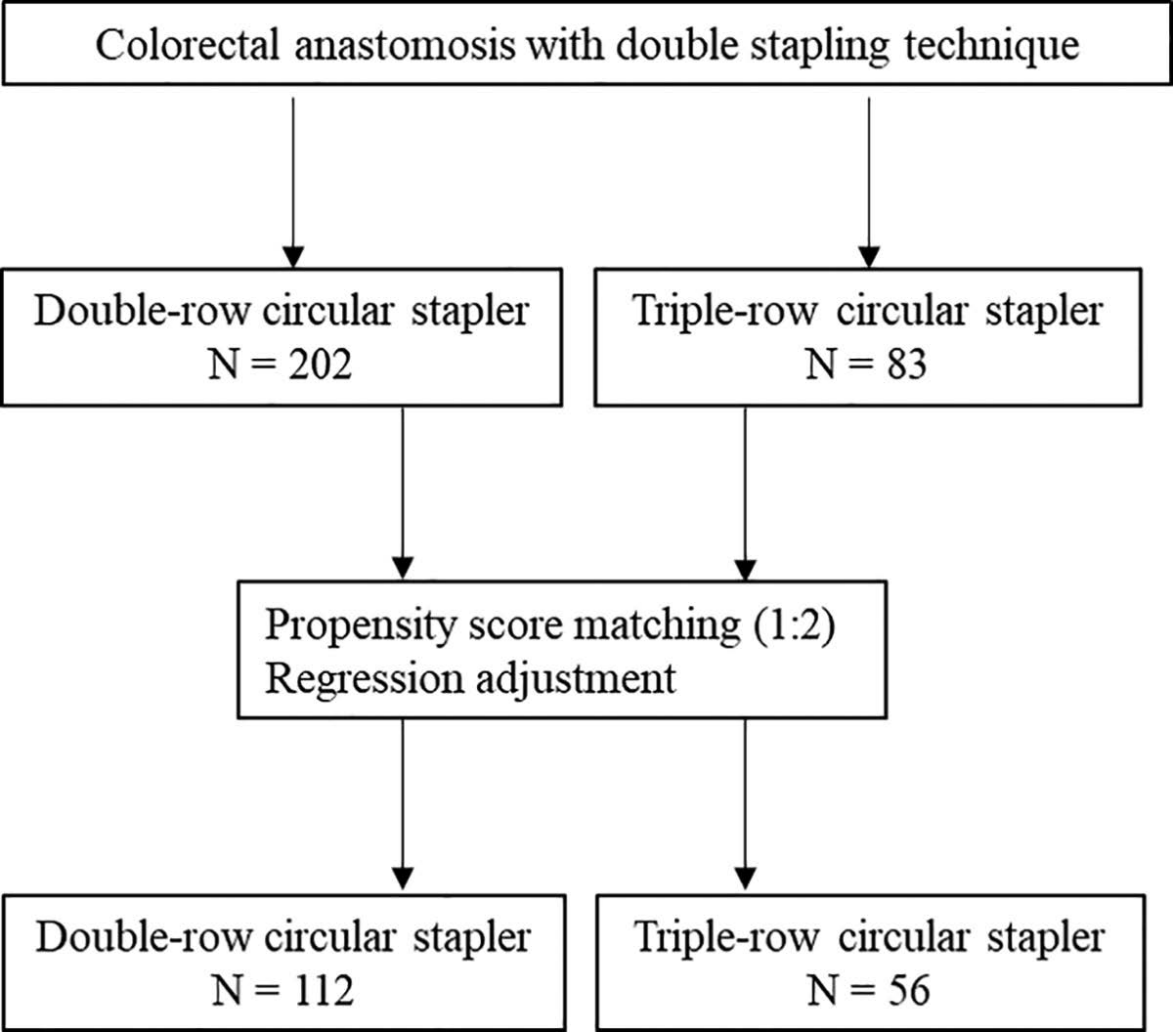
Flowchart of this study.

A total of 285 patients from our medical records who underwent colorectal surgery with left-sided circular stapled colorectal anastomosis above 5 cm from the anal verge with the DST at Tokyo Medical University Hospital between 2017 and 2021 were included in this retrospective study using a propensity score analysis. Patients aged ≥20 years who underwent colorectal anastomosis after left colectomy, sigmoidectomy, or anterior rectal resection for benign or malignant pathology were included, and those with anastomosis <5 cm from the anal verge (they did not have DST anastomosis) or who had preoperative therapy were excluded. Patients were divided into 2 groups according to the device used for the DST (DCS and TCS groups). A conventional DCS was used until December 2020. A novel TCS device (EEA™ circular stapler with Tri-Staple™ technology, 28 mm Medium/Thick, Covidien, New Haven, CT) was introduced in Japan in January 2021, and since then, our hospital has preferentially used this device. The flowchart of this study is shown in **Figure 1**. This study was approved by the institutional review board of the Tokyo Medical University Hospital.

### 2.2. Surgical treatment

No changes were made during the study period in perioperative care or surgical principles. The same group of dedicated colorectal surgeons performed all surgeries.

The anal side of the colorectum was incised with a linear stapler. The anvil of the circular stapler was secured in place, and end-to-end anastomosis was performed with the DST. The rod of the circular stapler was inserted from the opposite side of the linear staple line, piercing the rectal stump near the linear staple line. When the anvil and rod were combined, we waited 20 seconds before firing. After firing, the stapler was held for 10 seconds and then released. After anastomosis, we usually perform the observation of the anastomosis with the colonoscopy.

### 2.3. Postoperative care for AL

The incidence of AL within 30 days of surgery was recorded. AL was diagnosed according to the International Study Group of Rectal Cancer definition^[10]^ as a defect of the intestinal wall at the anastomotic site leading to a communication between the intra- and extraluminal compartments or as an abscess adjacent to the anastomosis. Computed tomography scanning with rectal contrast was performed in patients with suspected AL in the absence of unquestionable clinical signs of peritonitis, which would require urgent surgery. The following computed tomography findings were considered suggestive of anastomotic failure: contrast leakage from inside the bowel to the pelvis and/or abdominal cavity, and abscess and/or perianastomotic collection associated with or without localized pneumoperitoneum.

### 2.4. Statistical analysis

The primary outcome was the risk of AL by type of circular stapler used. Descriptive statistics were obtained for all variables. In the case of quantitative variables, normality was assessed using the Shapiro–Wilk test. Data were presented as median and range.

Absolute values and frequencies were calculated for qualitative variables. Then, possible relationships between quantitative and objective variables were investigated using parametric or nonparametric tests depending on variable distribution. Relationships among qualitative variables were tested using the chi-square test. Propensity score analysis was performed to adjust for heterogeneity between the DCS and TCS groups. Multivariate logistic regression was used to generate a propensity score predicting condition by a device (DCS or TCS). The following 11 covariates were included: age, sex, body mass index, American Society of Anesthesiologists (ASA) score, preoperative hemoglobin, surgical procedure (open or minimally invasive surgery), malignant disease (yes or no), diverting stoma (yes or no), operative time, anastomotic level from the anal verge (middle rectum (5–10 cm) and upper rectum (10–15 cm)), and postoperative complications (Clavien-Dindo classification >2). Each patient was assigned an estimated propensity score, which represented the patient’s predicted probability of the device selected. We specified matching IDs based on propensity scores and divided patients into 2 groups, pairing patients according to similarities in their characteristics. Then, 1:2 propensity score matching was performed. Each patient in the DCS group was matched to a patient in the TCS group who had the closest propensity score on the logit scale with a caliper of 0.2. Propensity scores were also used for regression adjustment, in which the treatment effect was estimated by adjusting for the impact of background covariates in a regression model. All statistical analyses were performed using SPSS software (IBM® SPSS® Statistics for Windows, Version 25.0; IBM, Chicago, IL) and EZR (Saitama Medical Center, Jichi Medical University, Saitama, Japan), a graphical user interface for R (The R Foundation for Statistical Computing, Vienna, Austria). The level for statistical significance was set at a *P* value of <.05.

## 3. Results

### 3.1. Patient and clinical characteristics

Baseline characteristics of the entire cohort are summarized in **Table 1**. The AL rate in the entire cohort was 6.0%. Patient characteristics of 2 groups are shown in **Table 2**. In the entire cohort, patients in the DCS group were significantly older compared to those in the TCS group (*P* = .01), and significantly fewer patients in the DCS group had a low ASA score compared to the TCS group (*P* < .001). No significant differences were observed in other covariates and AL rates between the 2 groups.

**Table 1.** Baseline characteristics.

Factor	
Age	62 (28– 96)
Sex	
Male	159 (55.8)
Female	126 (44.2)
BMI (kg/m^2^)	23.1 (13.1–37.7)
ASA score	
1	121 (42.5)
2	147 (51.6)
3	17 (6.0)
Preoperative Hb	12.4 (7.4–17.0)
Malignant disease	
No	28 (9.9)
Yes	256 (90.1)
Procedure	
Open	38 (13.4)
MIS	245 (86.6)
Diverting stoma	
No	229 (80.6)
Yes	55 (19.4)
Operative time (min)	268 (105–826)
Anastomosis level	
Middle rectum (5–10 cm)	86 (30.3)
Upper rectum (10–15 cm)	198 (69.7)
Postoperative complication (C-D > 2)	
No	254 (89.1)
Yes	31 (10.9)
Anastomotic leakage	
No	268 (94.0)
Yes	17 (6.0)
Anastomotic device	
DCS	202 (70.9)
TCS	83 (29.1)

Data are expressed as median (range) or n (%).

ASA = American Society of Anesthesiologists, BMI = body mass index, C-D = Clavien-Dindo classification, Hb = hemoglobin, MIS = minimally invasive surgery.

**Table 2.** Characteristics of both the entire cohort and the propensity score-matched pairs.

Factor	Entire cohort (n = 285)			1:2 propensity score–matched pairs (n = 118)		
	Device			Device		
	DCS (n = 202)	TCS (n = 83)	*P* value	DCS (n = 112)	TCS (n = 56)	*P* -value
Age	70 (33–96)	63 (28–87)	.01	68 (33–93)	69 (28–87)	.68
Sex						
Male	110 (54.5)	49 (59.0)	.51	65 (58.0)	31 (55.4)	.74
Female	92 (45.5)	34 (41.0)		47 (42.0)	25 (44.6)	
BMI, kg/m^2^	22.5 (14.5–37.7)	23.1 (13.1–36.1)	.12	22.9 (15.4–37.7)	22.8 (13.1–33.9)	.38
ASA score						
1	91 (45.0)	30 (36.1)	<.001	36 (32.1)	25 (44.6)	.002
2	111 (55.0)	36 (43.4)		76 (67.9)	27 (48.2)	
3	0 (0.0)	17 (20.5)		0 (0.0)	4 (7.1)	
Preoperative Hb	12.8 (7.8–17.0)	12.4 (7.4–17.0)	.77	12.7 (8.4–16.4)	12.7 (9.5–16.9)	.94
Malignant disease						
No	19 (9.5)	9 (10.8)	.83	8 (7.1)	3 (5.4)	.75
Yes	182 (90.5)	74 (89.2)		104 (92.9)	53 (94.6)	
Procedure						
Open	22 (10.9)	16 (19.8)	.06	13 (11.6)	5 (8.9)	.79
MIS	180 (89.1)	65 (80.2)		99 (88.4)	51 (91.1)	
Diverting stoma						
No	165 (82.1)	64 (77.1)	.33	88 (78.6)	44 (78.6)	1.00
Yes	36 (17.9)	19 (22.9)		24 (21.4)	12 (21.4)	
Operative time	251 (85 - 826)	269 (105–653)	.85	256 (85–826)	274 (105–653)	.86
Anastomosis level						
Middle rectum (5–10 cm)	56 (27.7)	30 (36.6)	.16	40 (35.7)	21 (37.5)	.87
Upper rectum (10–15 cm)	146 (72.3)	52 (63.4)		72 (64.3	5 (62.5)	
Postoerative complication (C-D > 2)						
No	176 (87.1)	78 (94.0)	.10	99 (88.4)	52 (92.9)	.43
Yes	26 (12.9)	5 (6.0)		13 (11.6)	4 (7.1)	
Anastomotic leakage						
No	187 (92.6)	81 (97.6)	.17	99 (88.4)	55 (98.2)	.04
Yes	15 (7.4)	2 (2.4)		13 (11.6)	1 (1.8)	

Data are expressed as median (range) or n (%).

ASA = American Society of Anesthesiologists, BMI = body mass index, C-D = Clavien-Dindo classification, DCS = double-row circular stapler, Hb = hemoglobin, MIS = minimally invasive surgery, TCS = triple-row circular stapler.

**Figure 2.**
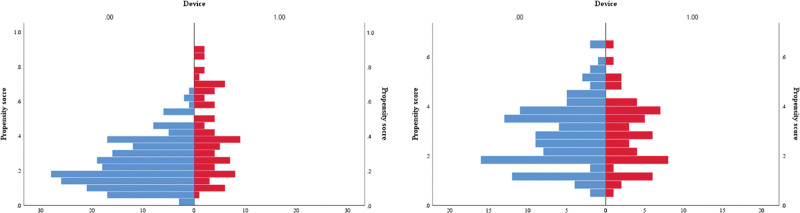
Distribution of propensity scores before and after case matching. (A) Distribution of propensity scores in the entire cohort. (B) Distribution of propensity scores in the 1:2 propensity score–matched cohort.

A 1:2 propensity score–matched analysis was performed according to propensity scores to adjust for heterogeneity in the DCS and TCS groups, with 11 covariates. Distributions of propensity scores before and after case matching are shown in **Figure 2**. Both DCS and TCS groups (DCS: 112 patients, TCS: 56 patients) showed a well-matched distribution with respect to patient and clinical characteristics in the adjusted analysis after case matching (**Table 2**). There were no significant differences in covariates other than ASA score between the 2 groups.

### 3.2. Anastomotic leakage

There are no critical stapler failures with intraoperative colonoscopy. AL was observed in a total of 17 patients (6.0%; **Table 1**). Before case matching, 15 patients (7.4%) in the DCS group and 2 patients (2.4%) in the TCS group (*P* = .17) had AL, with no significant difference between the 2 groups (**Table 2**). After case matching, 13 patients (11.6%) in the DCS group and 1 patient (1.8%) in the TCS group (*P* = .04) had AL, with the former showing a significantly higher proportion (**Table 2**). Among patients who had AL after case matching, there were 6 patients in the DCS group (46%) and no patients in the TCS group (0%) who had diverting stoma.

### 3.3. Regression adjustment including propensity scores

Regression models were created by applying propensity scores to adjust for group differences via regression adjustment. Odds ratios for AL in the DCS group versus the TCS group were 0.31 (95% confidence interval [CI]: 0.07–1.38) in the entire cohort, 0.15 (95% CI: 0.02–0.64) in the adjusted cohort, and 0.14 (95% CI: 0.02–1.09) in the 1:2 propensity score-matched cohort (**Table 3**).

**Table 3.** Hazard ratios to measure the effects of circular staplers.

Model	Sample size (number of patients)		Odds ratio (95% CI)
	DCS	TCS	
Unadjusted model	202	83	0.31 (0.07–1.38)
Propensity score–adjusted model			
Regression adjustment	202	83	0.15 (0.02–0.64)
Matching 1:2	112	56	0.14 (0.02–1.09)

CI = confidence interval, DCS = double-row circular stapler, TCS = triple-row circular stapler.

## 4. Discussion

Colorectal cancer is the second leading cause of cancer death in Japan. Surgical resection is the only curative treatment, with the laparoscopic approach now being used increasingly. However, AL is a major problem in patients undergoing laparoscopic low anterior resection, as this complication is associated with short-term and long-term outcomes such as local recurrence and patient survival.^[11–16]^ Reducing AL has been a constant challenge for colorectal surgeons. The DST, first reported by Knight and Griffen in 1980,^[17,18]^ is currently the most commonly accepted and widely used method for colorectal anastomosis after left-sided colorectal resection.^[19]^ However, despite technical improvements and advances in equipment, the rate of leakage after anastomosis with the DST remains at around 6% to 18%.^[14,19–23]^

Many factors contribute to AL after colorectal anastomosis with the DST, including tissue thickness, blood flow, ischemia, and tension.^[24,25]^ In particular, leakage pressure is considered the most important factor in assessing the quality of a fresh intestinal suture line.^[26]^ A greater leakage pressure is associated with a stronger anastomosis <1 week after surgery,^[27]^ suggesting that leakage pressure reflects the strength of the anastomosis.

TCS was developed to increase the strength of anastomosis. Since the EEA™ circular stapler, a new circular stapler with triple-row staples, was introduced in Japan in January 2021, this device has been preferentially used to perform the DST in our hospital. Although we expect that TCS is useful for creating a stronger anastomosis, no clinical results have been reported to support this. The present study is the first to compare clinical outcomes between TCS and DCS in colorectal anastomosis with the DST, and a propensity score analysis was used to minimize selection bias. After case matching, the rate of AL was significantly lower in the TCS group (1.8%) compared to the DCS group (11.6%), suggesting that TCS can markedly reduce the risk of AL. Moreover, we found a marked improvement relative to the previously reported AL rate.

The number of staples in TCS is increased by 50% compared to DCS, with no change in outer diameter. In addition, TCS has 3 rows of staples having different heights. These features may contribute to the reduced rate of AL for the following reasons. First, compared to DCS, which has a double row of staples of the same height, TCS allows for gradual compression from the inside to the outside of the lumen, gradually releasing pressure outward, thereby preventing severe compression damage.^[28–30]^ This mechanism may explain the greater pressure resistance of TCS. Simply tightening the conventional circular stapler may further press and crush the already tightened colon wall due to purse-string sutures and may thus result in the protrusion of a portion of the colon wall.^[31]^ Second, compression with staples of different heights may help maintain blood flow to the formed anastomosis.

This study has some limitations. First, we used a single-center nonrandomized clinical trial with historical controls in design. This study design introduces selection bias. In this study, the differences in background factors between the 2 groups were addressed to the extent possible using the statistical method of propensity score matching to minimize selection bias. However, due to selection bias, our results may overestimate the effect of TCS on reducing the risk of colorectal AL, compared to DCS. Second, patients who were operated on during different periods were included in this study (DCS: 2017–2020; TCS: 2021–present). However, during the 5-year study period, all operations were performed by the same surgical team, including experts in colorectal surgery. A prospective study will be needed to further clarify the effectiveness of TCS.

## 5. Conclusions

In conclusion, the present propensity score analysis suggests that the use of TCS contributes to a reduction in AL risk after colorectal anastomosis with the DST.

### Author contributions

J.M. wrote the manuscript. T.T., K.K., H.K., M.E., and T.I. acquired data for the study. All authors reviewed the manuscript. N.F., K.K., Y.N., and A.T. drafted the manuscript or revised it critically for important intellectual content.
